# Yindan Jiedu Granules, a Traditional Chinese Medicinal Formulation, as a Potential Treatment for Coronavirus Disease 2019

**DOI:** 10.3389/fphar.2020.634266

**Published:** 2021-02-05

**Authors:** Jingyuan Liu, Yuyong Jiang, Yao Liu, Lin Pu, Chunjing Du, Yuxin Li, Xiaojing Wang, Jie Ren, Wei Liu, Zhiyun Yang, Zhihai Chen, Rui Song, Wen Xie, Xianbo Wang

**Affiliations:** ^1^ Department of Critical Care Medicine, Beijing Ditan Hospital, Capital Medical University, Beijing, China; ^2^ Center of Integrative Medicine, Beijing Ditan Hospital, Capital Medical University, Beijing, China; ^3^ Department of Pharmacy, Beijing Shijitan Hospital, Capital Medical University, Beijing, China; ^4^ Center of Infectious Diseases, Beijing Ditan Hospital, Capital Medical University, Beijing, China; ^5^ Liver Diseases Center, Beijing Ditan Hospital, Capital Medical University, Beijing, China

**Keywords:** COVID-19, herbal medicine, Yindan Jiedu granules, recovery proportion, fever, pulmonary exudative lesions

## Abstract

**Background:** *Yindan* *Jiedu* Granules (YDJDG) have been newly prescribed as a Chinese herbal formula. This study aimed to compare the efficacy of YDJDG and lopinavir-ritonavir in the treatment of coronavirus disease 2019 (COVID-19).

**Methods:** Overall, 131 patients with COVID-19 were included in this study. In addition to standard care, 60 of these patients received YDJDG (YDJDG group) and 71 received lopinavir-ritonavir (lopinavir-ritonavir group). Propensity score matching (PSM) was used to match the characteristics of individuals in the two groups, while the Kaplan-Meier method was used to compare the proportion recovery observed.

**Results:** Cox analysis revealed that YDJDG and CD4 ≥ 660 cells/µL were independent predictive factors of proportion recovery. At baseline, disease types differed between the YDJDG and lopinavir-ritonavir treatment groups. Furthermore, no significant adverse effects or toxicities relevant to YDJDG were observed. The median recovery time was 21 days in the YDJDG group and 27 days in the lopinavir-ritonavir group. After PSM (1:1), 50 patient pairs, YDJDG vs. lopinavir-ritonavir, were analyzed. In the YDJDG group, the proportion of recovered patients was remarkably higher than that observed in the lopinavir-ritonavir group (*p* = 0.0013), especially for those presenting mild/moderate disease type and CD4 < 660 cells/µL. In the YDJDG group, the mean duration of fever and pulmonary exudative lesions was significantly shorter than that observed in the lopinavir-ritonavir group (*p* = 0.0180 and *p* = 0.0028, respectively).

**Conclusion:** YDJDG reveals the potential to hasten the recovery period in COVID-19 patients with mild/moderate disease type or CD4 < 660 cells/µL by shortening the mean duration of fever and pulmonary exudative lesions.

## Introduction

Currently, coronavirus disease-2019 (COVID-19) is an international public health emergency. Patients with severe disease type account for approximately 15% of the cases (Wu and McGoogan. 2020); hence, there is an urgent need for effective drugs. At present, no specific and effective antiviral treatment is available for the treatment of COVID-19. Optimized supportive care remains the mainstay of therapy. Antivirals investigated for the treatment of COVID-19 in clinical trials or *in vitro* studies include inhibitors of viral RNA polymerase/RNA synthesis (remdesivir), inhibitors of viral protein synthesis (lopinavir-ritonavir), viral entry inhibitors (chloroquine), and immunomodulators (Nitazoxanide) (Şimşek Yavuz and Ünal. 2020). Among patients hospitalized for severe COVID-19, 68% of patients treated with remdesivir experience clinical improvement. Hence, a randomized, placebo-controlled trial is required to measure the efficacy of remdesivir therapy (Grein et al., 2020). In a controlled, randomized, open-label trial, lopinavir-ritonavir treatment has failed to demonstrate an improvement over standard treatment in adult patients with severe COVID-19 (Cao et al., 2020). Furthermore, there is insufficient evidence to determine whether chloroquine/hydroxychloroquine are safe and effective treatments for COVID-19 (Gbinigie and Frie, 2020). Moreover, at least 80 trials investigating chloroquine or hydroxychloroquine have been registered worldwide to provide improved clinical guidance.

Previous studies have demonstrated that a combination of Chinese and Western medicine is more effective in shortening the duration of ventilator use, improving lung infiltration, and shortening the length of hospital stay when compared with Western medicine alone (Ren et al., 2004; Wang et al., 2012). Lianhua Qingwen Granule (LHQWG) was used to treat influenza and demonstrated superior efficacy to oseltamivir (Zhao et al., 2014). With the progression of the COVID-19 pandemic, a growing number of treatment plans released by the National Health Commission of the People’s Republic of China (NHC) emphasize that patients with COVID-19 can be treated with integrated traditional Chinese and Western medicine at all disease stages (Jin et al., 2020). In China, more than 85% of patients with COVID-19 have received traditional Chinese medicine (TCM) treatment. In the LHQWG group, the symptom recovery rate was significantly higher than that in the control group (Hu et al., 2020). One retrospective study has shown that Jin-hua Qing-gan granules can effectively shorten the duration of nucleic acid detection and promote the absorption of the inflammatory exudate in pneumonia, presenting no obvious adverse reactions in patients with COVID-19 (Liu Y et al., 2020). Furthermore, the application of TCM in the treatment of severe COVID-19 can reportedly shorten the average hospital stay, as well as the nucleic acid conversion time, by more than 2 days (Chan et al., 2020).

*Yindan Jiedu* Granules (YDJDG), prescribed as a newly applied Chinese herbal formula at the Beijing Ditan Hospital, has demonstrated beneficial effects in COVID-19. To further evaluate the efficacy of oral YDJDG in the treatment of COVID-19, we conducted a prospective, single-center cohort study, utilizing lopinavir-ritonavir as the control drug.

## Materials and Methods

### Patients

**FIGURE 1:**
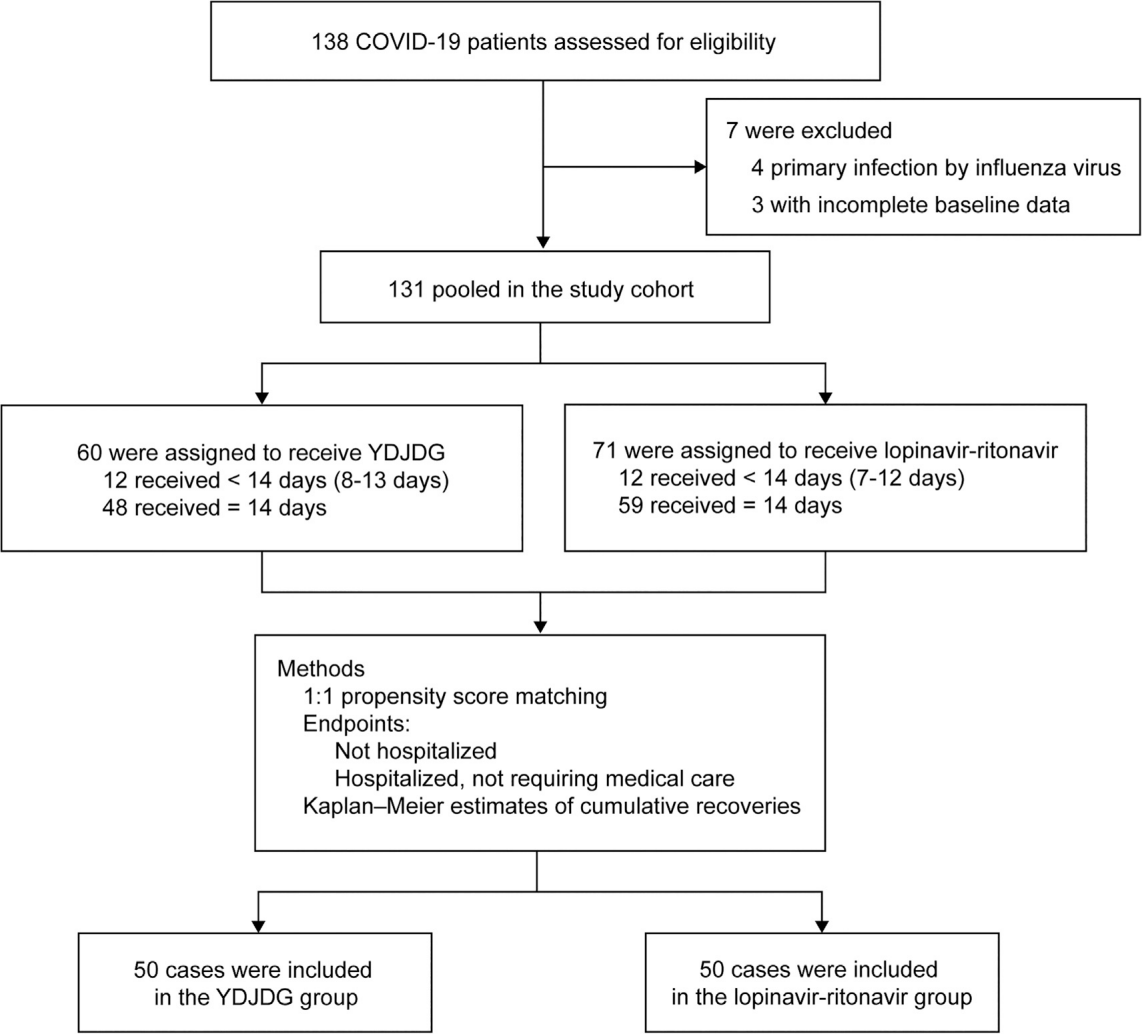
Study flow chart. YDJDG, *Yindan Jiedu* granules; COVID-19, Coronavirus disease-2019.

Between January 29, 2020, and March 21, 2020, 138 patients with COVID-19 admitted to Beijing Ditan Hospital were enrolled in this study. The diagnosis, clinical classification, inclusion, and exclusion criteria have been described in a paper previously published by our research group (Liu Z et al., 2020). Collectively, 131 patients diagnosed with COVID-19 and receiving YDJDG or lopinavir-ritonavir therapy comprised the research cohort. Among these, 60 were receiving YDJDG therapy and 71 were receiving lopinavir-ritonavir (Figure 1). This investigation was approved by The Institutional Research Ethics Committee affiliated with the Beijing Ditan Hospital, Capital Medical University (Beijing, China). All patients provided a signed informed consent form.

### Interventional Medicine

Patients receiving YDJDG or lopinavir-ritonavir came from two wards. YDJDG is a herbal extract composed of *Ephedrae Herba*, *Gypsum Fibrosum*, *Mori Cortex*, *Scutellariae Radix*, *Lepidii Semen*, *Lonicerae Japonicae Flos*, *Scrophulariae Radix*, *Moutan Cortex*, *Rehmanniae Radix*, *Atractylodis Macrocephalae Rhizoma*, and *Cimicifugae Rhizoma* (Table 1). YDJDG was produced as follows: *Gypsum Fibrosum* (420 g) was decocted with water for 20 min, with the other ten ingredients added to the decoction twice, for 1 h each time; then, the filtrates were combined. Under reduced pressure (-0.06 mp-0.08 mp, 70–90°C), the filtrate was concentrated to a thick paste, with a relative density of 1.10–1.20 (60°C). Using a specific ratio of thick paste and dextrin (2:1), 1,000 g granules were prepared. YDJDG has been approved by the Beijing Medical Products Administration (China) (No. Z20200012000). The dose of YDJDG was 12 g or 24 g, to be administered orally three times per day; the dose of lopinavir-ritonavir was 400–100 mg, administered orally twice daily (Cao et al., 2020). Patients discharged within 14 days received YDJDG or lopinavir-ritonavir until discharge, and patients discharged after 14 days were treated with YDJDG or lopinavir-ritonavir for a total of 14 days (Figure 1).

**TABLE 1:** The ingredients contained in YDJDG.

Latin name	Source species	Picture	Weight (g)
*Ephedrae Herba*	*Ephedra sinica* Stapf	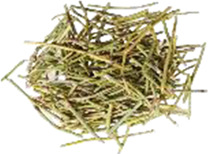	84
*Gypsum Fibrosum*	*Gypsum*	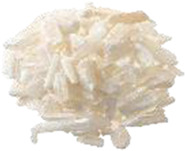	420
*Mori Cortex*	*Morus alba* L.	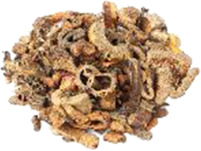	210
*Scutellariae Radix*	*Scutellaria baicalensis* Georgi	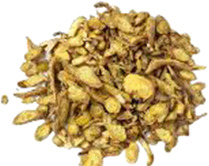	210
*Lepidii Semen*	*Lepidium apetalum* Willd.	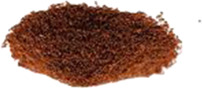	280
*Lonicerae Japonicae Flos*	*Lonicera japonica* Thunb.	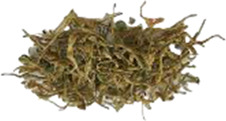	210
*Scrophulariae Radix*	*Scrophularia ningpoensis* Hemsl.	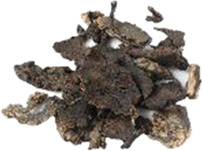	210
*Moutan Cortex*	*Paeonia suffruticosa* Andrews	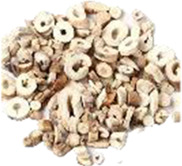	210
*Rehmanniae Radix*	*Rehmannia glutinosa* (Gaertn.) DC.	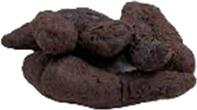	210
*Atractylodis Macrocephalae Rhizoma*	*Atractylodes macrocephala* Koidz.	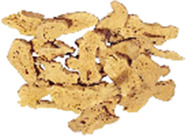	210
*Cimicifugae Rhizoma*	*Actaea heracleifolia* (Kom.) J.Compton	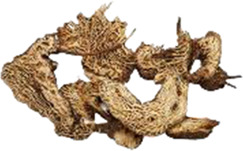	210

The standard care included supplemental oxygen, noninvasive and invasive ventilation, and nebulization inhalation treatment. In the YDJDG group, 19 (31.7%) patients received oxygen support and 2 (3.3%) received noninvasive ventilation. In the lopinavir-ritonavir group, 21 (29.6%) patients received oxygen support and 1 (1.4%) received noninvasive ventilation.

### Observation Outcome

The main outcome was the recovery time, defined as the period from the first day of enrollment to discharge or hospitalization without oxygen supplementation and continuous medical care. The secondary outcomes included the time to symptom improvement, including the return of body temperature to normal for at least 3 days, significantly improved cough, significantly absorbed pulmonary inflammation, and negative nucleic acid test results obtained twice consecutively (at intervals of 24 h).

### Statistical Analysis

Age and days from illness onset to admission time are expressed as median (range), categorical variables are expressed as number (%), and continuous variables are expressed as median (interquartile interval) or mean ± standard deviation. The cut-off values for continuous variables were calculated based on mean values. The differences between the two groups were determined using the *t*-test for continuous variables presenting normal distribution; the Mann-Whitney *U* test was applied for continuous variables that did not have a normal distribution, with the Chi-square test used for count data. Cox regression analysis was performed to identify independent factors of recovery rate. A *p* value of < 0.05 was considered statistically significant. Data analyses were performed using SPSS 22.0 statistical package (SPSS, Inc., Chicago, IL, United States). To reduce bias in the analysis, a one-to-one propensity score matching (PSM) was used. The YDJDG group was matched with the lopinavir-ritonavir group according to the generated propensity scores using a caliper width of 0.05. The Kaplan-Meier method was used to compare proportion recovery. PSM and Kaplan-Meier analyses were performed using the R software version 3.6.3 (R Foundation for Statistical Computing, Vienna, Austria).

### Sample and Chemicals

Three bags of Chinese medicine granules were randomly selected and weighed accurately about 200 mg. Methanol:water (8:2, V:V) was added in the ratio of 1:5 (1 g:5 ml), and the mixture was vortex-mixed. Centrifugation was performed at 4°C for 10 min with a centrifugal force of 20,000 × g. The supernatant was filtered with 0.22 um membrane, and the filtrate was taken for analysis. Chemicals of phenylalanine, berberine, ferulic acid, quercetin, and baicalein were purchased from Solarbio (Beijing, China). Arachidonic acid was purchased from Shanghai Yuanye Bio-Technology Co., Ltd (Shanghai, China). Wogonin was purchased from National Institutes for Food and Drug Control (Beijing, China). The details were shown in Table 2.

**TABLE 2:** Seven chemicals found in YDJDG.

Chemicals	CAS	Molecular formula	Molecular weight	Charged properties	MS1	MS2	RT
Phenylalanine	63-91-2	C9H11NO2	165.19	+	166.08611	120.08081	4.15
Berberine	2086-83-1	C20H18NO4	336.12	+	336.12219	320.09122	5.87
Ferulic acid	1135-24-6	C10H10O4	194.19	+	195.06490	177.0545	6.59
Quercetin	117-39-5	C15H10O7	302.00	+	303.04910	153.0181	7.40
Baicalein	491-67-8	C15H10O5	270.24	+	271.05927	123.00768	7.82
Wogonin	632-85-9	C16H12O5	284.26	+	285.07474	270.05157	8.14
Arachidonic acid	506-32-1	C20H32O2	304.23	+	287.00000	271.05511	8.20

### Liquid Chromatography-Tandem Mass Spectrometry (LC-MS-MS)

Liquid chromatography analysis was performed on Thermo UltiMate 3000 RS liquid chromatography system (Shanghai, China). The column was maintained at 35°C. The mobile phase consisted of water with 0.1% formic acid and acetonitrile with 0.1% formic acid. The column was eluted at a flow rate of 0.30 ml/min using a linear gradient as follows: 98% water phase ratio at 0–1 min, 80% water phase ratio at 20 min, 50% water phase ratio at 10 min, 20% water phase ratio at 15 min, 5% water phase ratio at 20–25 min, and 98% water phase ratio at 26–30 min.

Mass spectrometry was performed on an Thermo TSQ Quantum mass spectrometer (Shanghai, China). Tandem MS analyses were performed in ESI mode. Other parameters are as follows: detection method: full mass/dd-MS2; resolution: 70,000 (full mass); 17,500 (dd-ms2); scan range: 150.0–2000.0 m/z. The analysis was performed under positive and negative ion switching mode with a spray voltage of 3.8 kV (positive). High purity nitrogen was used as the sheath gas and auxiliary gas. The capillary temperature was maintained at 300°C.

## Results

### Baseline Characteristic

**FIGURE 2:**
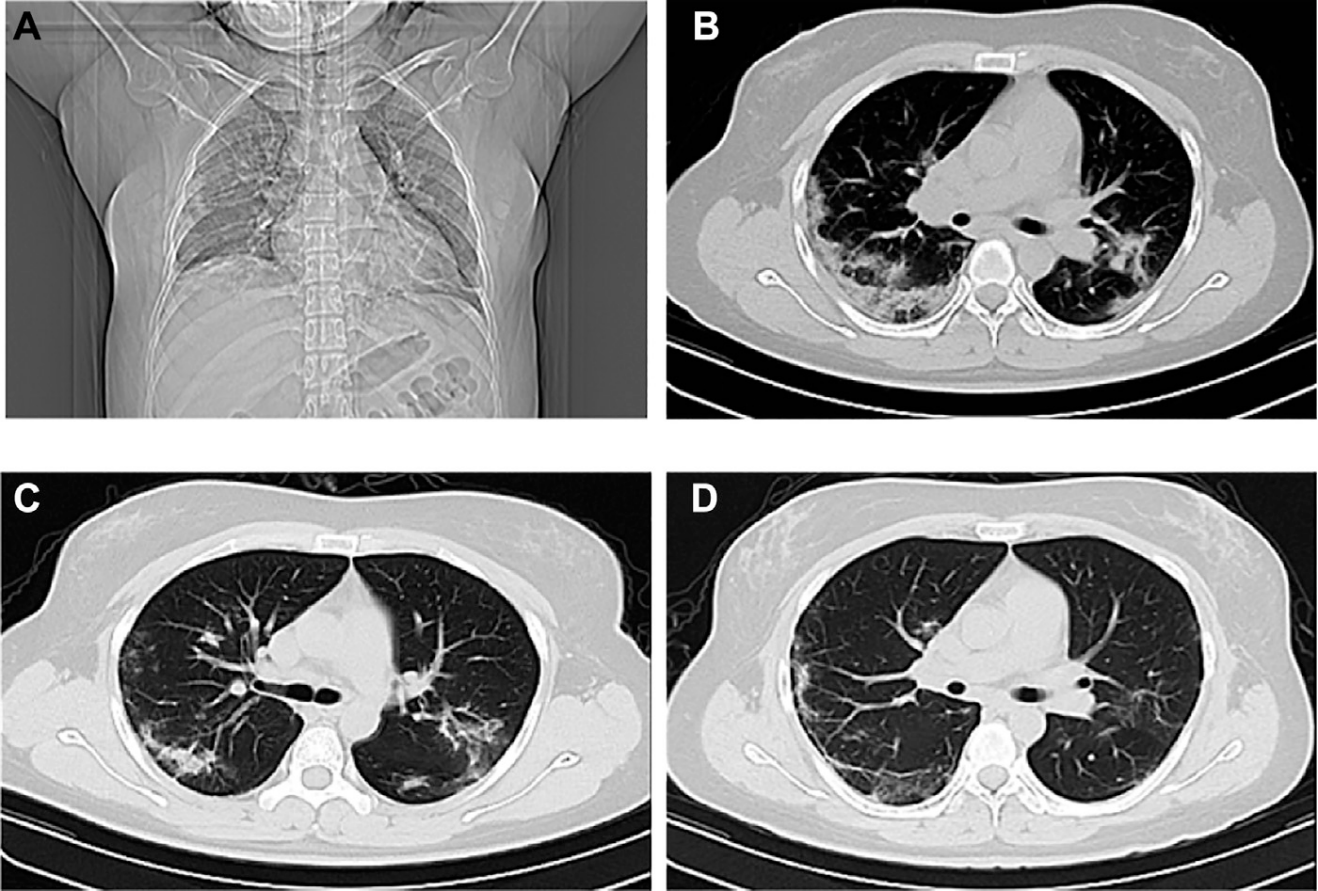
Computed tomography images of the chest before and after treatment. A 45-year-old female who presented with bilateral pulmonary infiltrates at treatment initiation with YDJDG in coronal plane **(A)** and transverse plane **(B)**; Marked absorption of bilateral pulmonary infiltrates at day 7 of YDJDG treatment **(C)**; Dissipation of lesions at day 14 of YDJDG treatment **(D)**. YDJDG: *Yindan Jiedu* Granules.

No significant difference was observed between the YDJDG and lopinavir-ritonavir groups in terms of age, gender, comorbidity, and laboratory tests, including neutrophil count, lymphocyte count, C-reactive protein, and CD4 cell count. In the YDJDG group, the median time from onset to admission was 5 days, similar to that observed in the lopinavir-ritonavir group. Furthermore, in the YDJDG group, 12 (20.0%) of 60 patients with COVID-19 were diagnosed with mild disease, 37 (61.7%) were diagnosed with moderate disease, 4 (6.7%) were diagnosed with severe disease, and 7 (11.7%) were diagnosed with critical disease. In the lopinavir-ritonavir group, 3 (4.2%) were diagnosed as mild, 58 (81.7%) were diagnosed as moderate, 9 (12.7%) were diagnosed as severe, and 1 (1.4%) was diagnosed as critical. Overall, 37 (61.7%) and 54 (76.1%) patients presented multiple or bilateral lung lobe involvements in the YDJDG and lopinavir-ritonavir groups, respectively (*p* = 0.075) (Table 3). Figure 2 presents the CT images of a typical patient receiving YDJDG therapy at initiation and after 7 and 14 days of treatment.

**TABLE 3:** Baseline characteristics of COVID-19 patients before and after propensity score matching.

	Before propensity score matching			After propensity score matching		
Variables	YDJDG	Lopinavir-Ritonavir	*p* value	YDJDG	Lopinavir-Ritonavir	*p* value
	(n = 60)	(n = 71)		(n = 50)	(n = 50)	
Median age (range)	41 (6–84)	41 (18–86)	0.982^a^	41 (6–84)	41 (18–82)	0.730^a^
Gender (M/F)	30/30	38/33	0.688^b^	24/26	28/22	0.423^c^
Median days from illness onset to admission time (range)	5 (1–38)	5 (0–29)	0.838^c^	5 (1–38)	4 (0–15)	0.530^b^
Coexisting comorbidity						
Hypertension	11 (18.3)	12 (16.9)	0.830^b^	9 (18.0)	8 (16.0)	0.790^c^
Diabetes	2 (3.3)	3 (4.2)	0.791^b^	2 (4.0)	1 (2.0)	0.558^c^
Cardiovascular disease	4 (6.7)	2 (2.8)	0.294^b^	3 (6.0)	2 (4.0)	0.646^c^
Chronic obstructive pulmonary disease	1 (1.7)	2 (2.8)	0.661^b^	1 (2.0)	1 (2.0)	1.000^c^
No. of coexisting comorbidities						
None	45 (75.0)	57 (80.3)	0.468^b^	37 (74.0)	41 (82.0)	0.334^c^
One	12 (20.0)	10 (14.1)	0.367^b^	11 (22.0)	6 (12.0)	0.183^c^
Two	3 (5.0)	4 (5.6)	0.872^b^	2 (4.0)	3 (6.0)	0.646^c^
Disease type						
Mild	12 (20.0)	3 (4.2)	0.005^b^	4 (8.0)	3 (6.0)	0.695^c^
Moderate	37 (61.7)	58 (81.7)	0.011^b^	37 (74)	44 (88.0)	0.074^c^
Severe	4 (6.7)	9 (12.7)	0.252^b^	4 (8.0)	2 (4.0)	0.400^c^
Critical	7 (11.7)	1 (1.4)	0.015^b^	5 (10.0)	1 (2.0)	0.092^c^
Neutrophil count, × 10⁹/L	3.1 (2.1–4.1)	3.2 (2.3–4.1)	0.773^c^	2.6 (1.0–3.9)	2.9 (2.3–3.8)	0.422^b^
Lymphocyte count, × 10⁹/L	1.3 (0.9–1.8)	1.2 (1.0–1.7)	0.794^c^	1.3 (0.9–1.8)	1.2 (1.0–1.6)	0.428^b^
NLR	2.4 (1.6–3.3)	2.6 (1.7–3.9)	0.759^c^	2.3 (1.5–3.1)	2.6 (1.8–3.5)	0.329^b^
C-reactive protein, mg/L	8.5 (1.4–18.6)	11.4 (2.6–28.5)	0.712^c^	11.1 (1.5–18.6)	10.6 (2.4–29.0)	0.869^b^
CD4, cells/ul	621.1 ± 206.1	680.1 ± 159.9	0.067^a^	605.1 ± 218.0	628.7 ± 111.6	0.498^b^
Multiple lung lobe or bilateral involvement	37 (61.7)	54 (76.1)	0.075^b^	34 (68.0)	40 (80.0)	0.147^c^

Data are presented as median (range), n (%), median (interquartile range), or means ± SD.

^a^ *p* values comparing YDJDG group and lopinavir-ritonavir group are from t-test.

^b^ Mann-Whitney U test.

^c^ χ^2^ test.

COVID-19, 2019 coronavirus disease; YDJDG, Yindan Jiedu Granules; NLR, neutrophil-to-lymphocyte ratio.

Compared to the lopinavir-ritonavir group, the YDJDG group exhibited an explicit and comparable difference in baseline characteristics before PSM. In terms of disease type, the YDJDG group had a higher number of patients with mild disease type and critical disease type (*p* = 0.005 and *p* = 0.015, respectively) and a lower number of patients with moderate type than the lopinavir-ritonavir group (*p* = 0.011). In the YDJDG vs. lopinavir-ritonavir group matched 1:1, the propensity score model was composed of variables, such as mild, moderate, and critical disease types. Following PSM, the crucial relevant characteristics demonstrated a balanced performance (Table 3).

### Proportion Recovery Analysis

**FIGURE 3:**
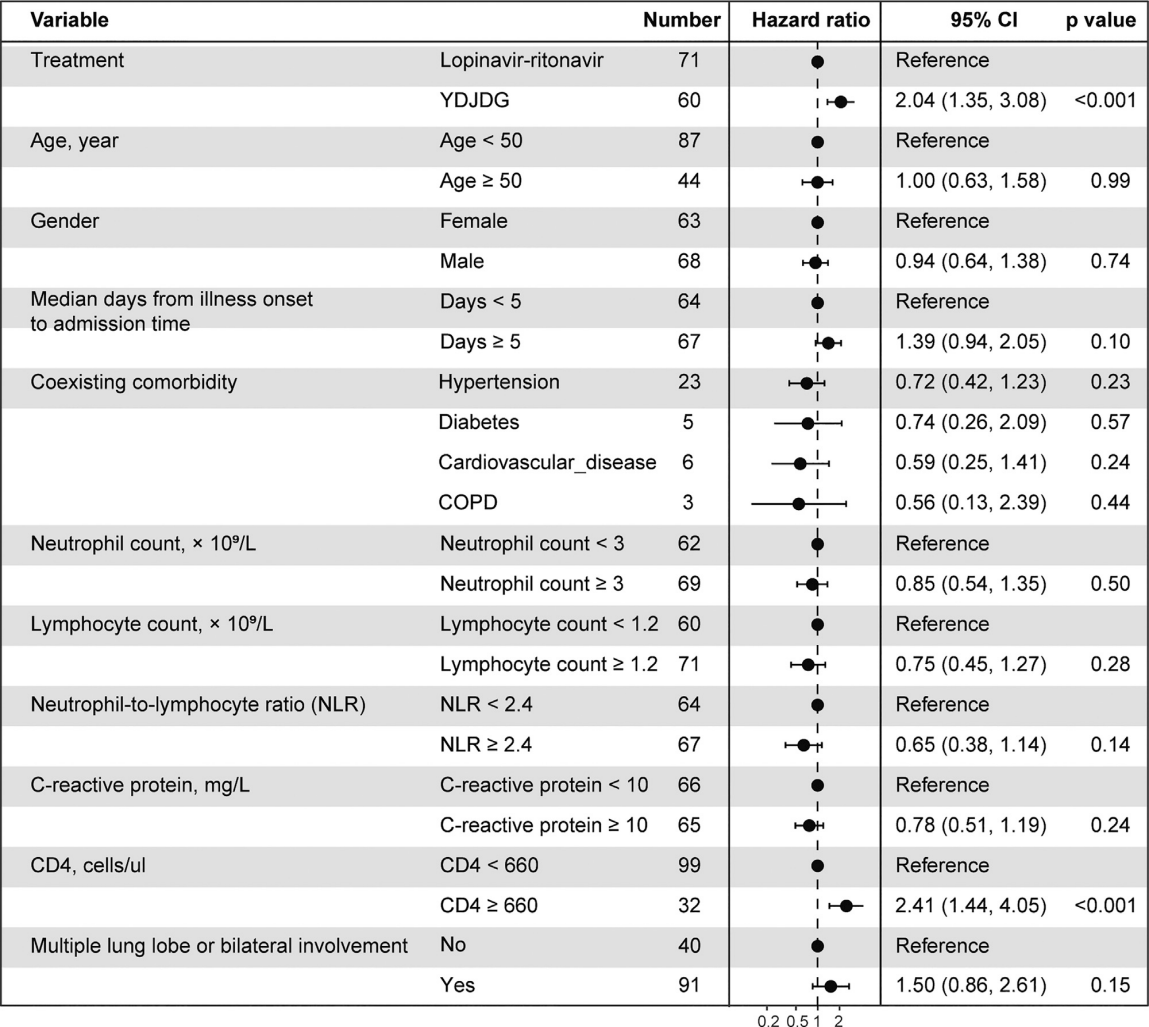
Cox regression analyses for proportion recovery in patients with COVID-19. YDJDG therapy and CD4 ≥ 660 cells/µL are significant factors affecting recovery (*p* < 0.001 for both). COVID-19, Coronavirus disease-2019; YDJDG, *Yindan Jiedu* granules.

Based on the findings of the Cox regression analysis, YDJDG therapy and CD4 ≥ 660 cells/µL were found to be significant factors affecting the prognosis for recovery (*p* < 0.001 for both) (Figure 3).

**FIGURE 4:**
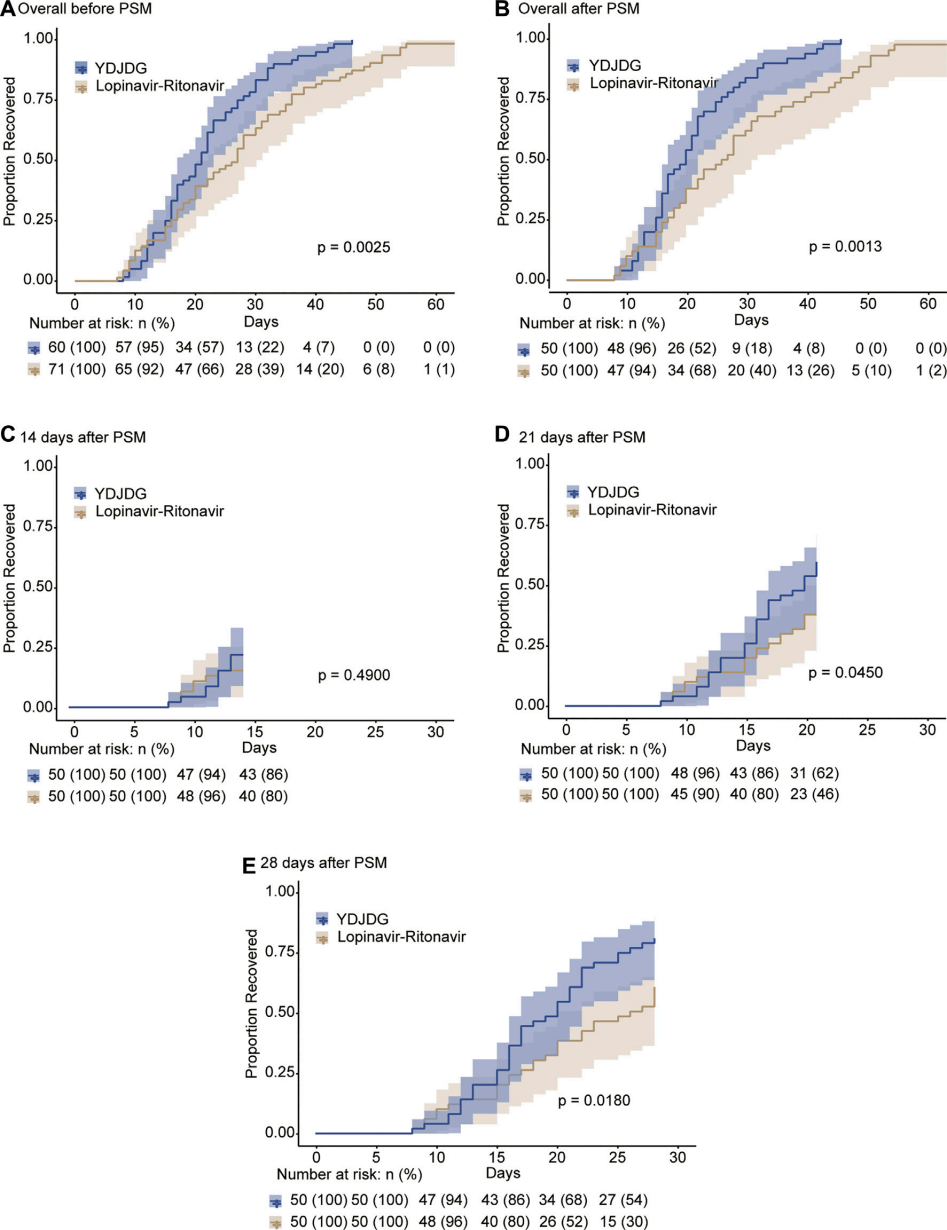
Kaplan-Meier estimates of proportion recovery. Proportion recovery estimates are shown in all patients before PSM **(A)** and after PSM **(B)**, after a follow-up of 14 days **(C)**, after a follow-up of 21 days **(D)**, and after a follow-up of 28 days **(E)**. The median and 95% confidence interval (95% CI) are presented in the figure.

The median recovery time was 21 days (interquartile range: 18–28 days) in the YDJDG group and 27 days (interquartile range: 16–36 days) in the lopinavir-ritonavir group (*p* = 0.009). Before PSM, the Kaplan-Meier analysis revealed that the YDJDG and lopinavir-ritonavir groups demonstrated a significant difference in the proportion recovery (*p* = 0.0025) (Figure 4A). PSM was used to form 50 pairs of patients in the YDJDG vs. the lopinavir-ritonavir group. Compared with the lopinavir-ritonavir group, the proportion recovery demonstrated a dramatic value in the YDJDG group (*p* = 0.0013) (Figure 4B). Furthermore, at 14, 21, and 28 days, the proportion recovery was compared between the two groups after PSM, and the results revealed a significant difference in proportion recovery in the 21- and 28-day analysis (*p* = 0.0450 and *p* = 0.0180, respectively) (Figures 4C–E).

**FIGURE 5:**
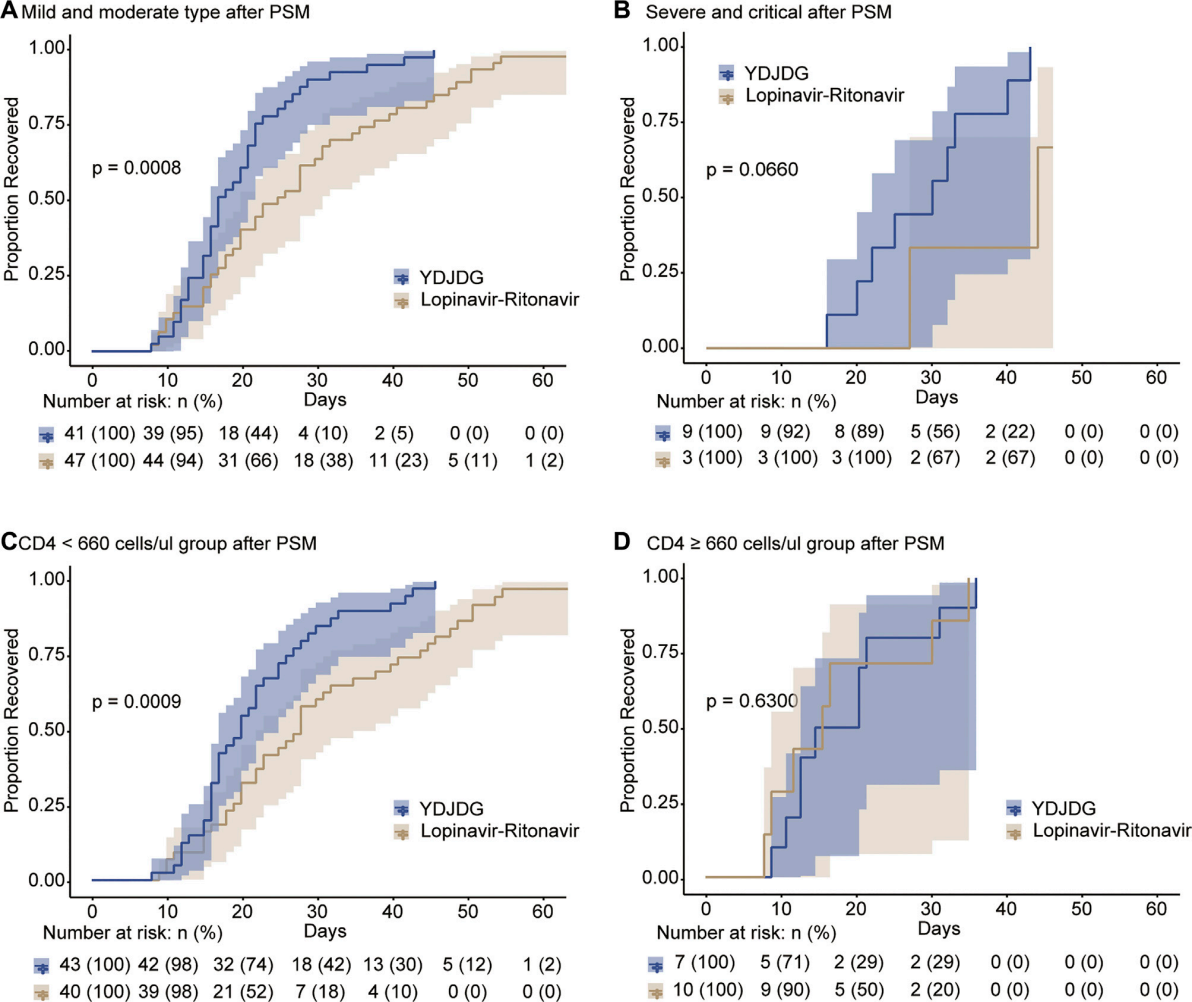
Kaplan-Meier estimates of proportion recovery in the subgroups. Proportion recovery estimates are shown for the mild/moderate disease type **(A)** and severe/critical disease type **(B)**, in the CD4 < 660 cells/µL **(C)** and CD4 ≥ 660 cells/µL **(D)** subgroups.

Following PSM, further analysis was performed to determine the proportion of patients who recovered in the mild/moderate disease type, severe/critical disease type, CD4 < 660 cells/µL, and CD4 ≥ 660 cells/µL subgroups, based on YDJDG or lopinavir-ritonavir therapy. Our findings revealed that the proportion recovery was significantly higher in those presenting mild/moderate disease type (Figure 5A) and CD4 < 660 cells/µL (Figure 5C) following YDJDG therapy (*p* = 0.0008 and *p* = 0.0009, respectively). Notably, the proportion recovery was similar for the severe/critical disease type (Figure 5B) and CD4 ≥ 660 cells/µL (Figure 5D) after YDJDG or lopinavir-ritonavir therapy (*p* = 0.0660 and *p* = 0.6300, respectively).

### Secondary Outcome Analysis

**FIGURE 6:**
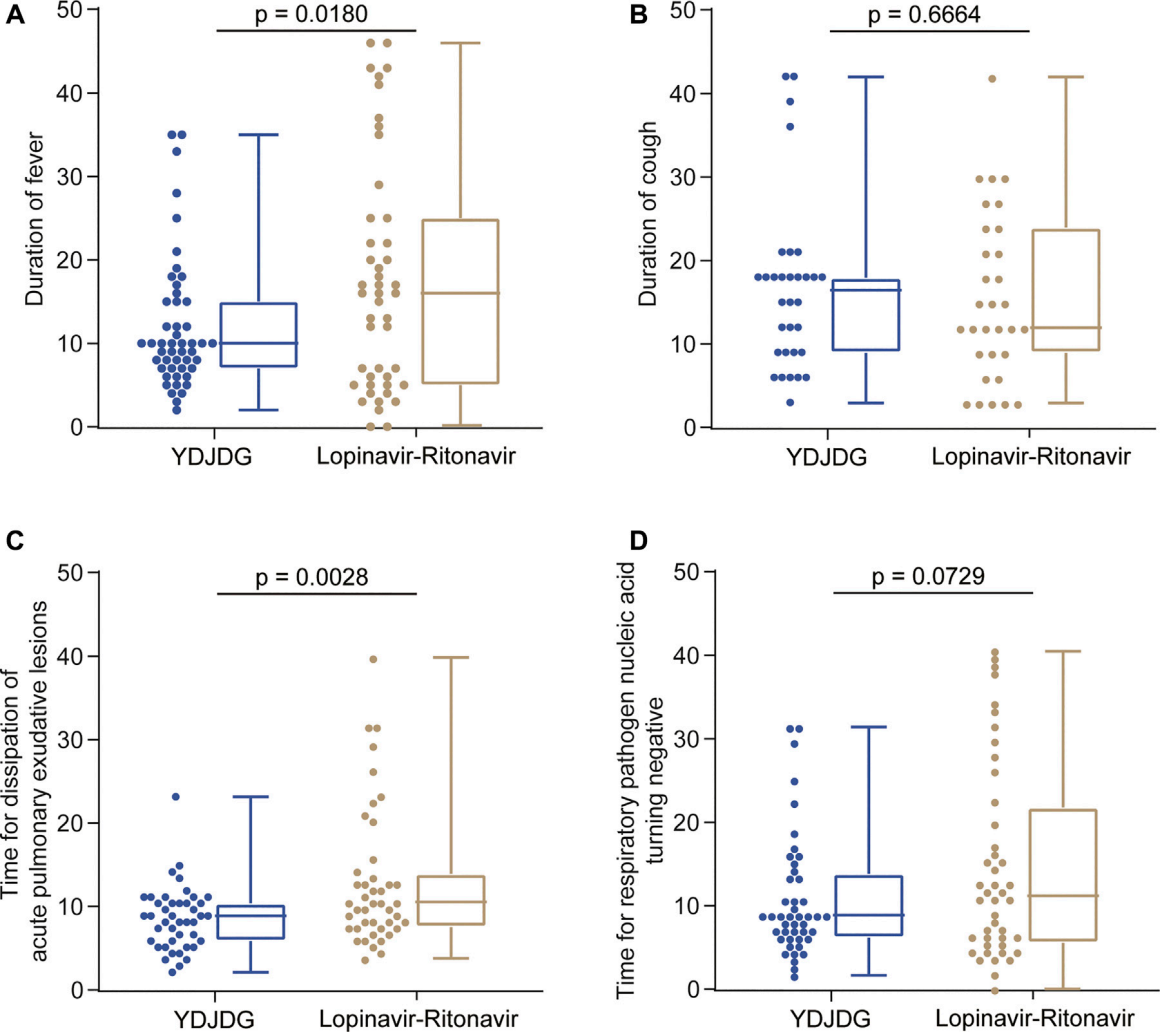
Duration of symptom recovery. Comparison of the duration of fever in the YDJDG and lopinavir-ritonavir groups **(A)**, the duration of cough in the YDJDG and lopinavir-ritonavir groups **(B)**, duration for dissipation of acute pulmonary exudative lesions **(C)**, and duration for the nucleic acid test to turn negative **(D)**.

Among the 50 patients in the YDJDG group, 32 (64.0%) presented with fever, 32 (64.0%) had a cough, and 46 (92.0%) revealed pulmonary inflammation in the imaging examination. In the lopinavir-ritonavir group, 38 (76.0%) had a fever, 29 (58.0%) presented a cough, and 47 (94.0%) demonstrated pulmonary inflammation in the imaging performed. In both groups, all patients were nucleic acid positive. Regarding fever and pulmonary exudative lesions, the mean durations were reported as 4.2 days (range: 1–13 days) and 11.6 days (range: 3–31 days), respectively, in the YDJDG group, which were significantly shorter than the 6.4 days (range: 1–43 days) and 17.2 days (range: 5–53 days) reported for the lopinavir-ritonavir group (*p* = 0.0180 and *p* = 0.0028, respectively) (Figures 6A,C). In both groups, no significant difference was observed between the mean duration of cough and positive nucleic acid results (*p* = 0.6664 and *p* = 0.0729, respectively) (Figures 6B,D).

### Safety Outcomes

During treatment, YDJDG was evaluated for safety, and liver and kidney functions were monitored before and 2 weeks after drug therapy (Table 4). For the liver function test, at enrollment, alanine aminotransferase was 24.6 (11.9–38.0) U/L, total bilirubin (TBIL) was 10.1 (7.2–13.5) µmol/L, and albumin was 40.0 (34.8–43.0) g/L. Following 2 weeks of YDJDG therapy, alanine aminotransferase was 24.3 (15.4–50.0) U/L, TBIL was 12.5 (8.0–23.6) µmol/L, and albumin was 37.4 (35.9–45.6) g/L. No significant difference was observed in liver function before and 2 weeks after treatment (*p* > 0.05 for all). Reportedly, two patients presented TBIL values greater than 18.8 μmol/L before treatment, and three patients presented TBIL values greater than 18.8 μmol/L after 2 weeks of YDJDG; in all these patients, the values returned to normal 2 weeks after drug withdrawal. At the time of admission, urea nitrogen and creatinine were 4.3 (3.5–4.2) mmol/L and 68.7 (55.1–81.4) µmol/L, respectively, and they were 4.3 (3.8–5.2) mmol/L and 66.6 (57.5–77.9) µmol/L, respectively, following YDJDG therapy for 2 weeks (*p* > 0.05 for both). At the time of enrollment, seven patients demonstrated a urinary protein of 1 + or above. Two weeks after enrollment, routine urine examination revealed that urinary protein was negative in six patients and did not change in one patient. Additionally, one patient presented with headache, one patient experienced dizziness, two patients complained of abdominal pain, four patients experienced diarrhea, and three patients reported a loss of appetite after YDJDG therapy; all symptoms resolved after the treatment course ended.

**TABLE 4:** YDJDG were evaluated for safety during treatment.

Adverse events	Before treatment	After treatment	*p* value
Liver function			
Alanine aminotransferase	24.6 (11.9–38.0)	24.3 (15.4–50.0)	0.589
Total bilirubin	10.1 (7.2–13.5)	12.5 (8.0–23.6)	0.054
Albumin	40.0 (34.8–43.0)	37.4 (35.9–45.6)	0.930
Renal function			
Urea nitrogen	4.3 (3.5–4.2)	4.3 (3.8–5.2)	0.633
Creatinine	68.7 (55.1–81.4)	66.6 (57.5–77.9)	0.941
Urine protein positive	7/60 (11.7%)	1/60 (1.7%)	0.028
Headache	0	1/60 (1.7%)	0.315
Dizzy	0	1/60 (1.7%)	0.315
Abdominal pain	0	2/60 (3.3%)	0.154
Diarrhea	0	4/60 (6.7%)	0.042
Loss of appetite	0	3/60 (5.0%)	0.079

### Sample Analysis

**FIGURE 7:**
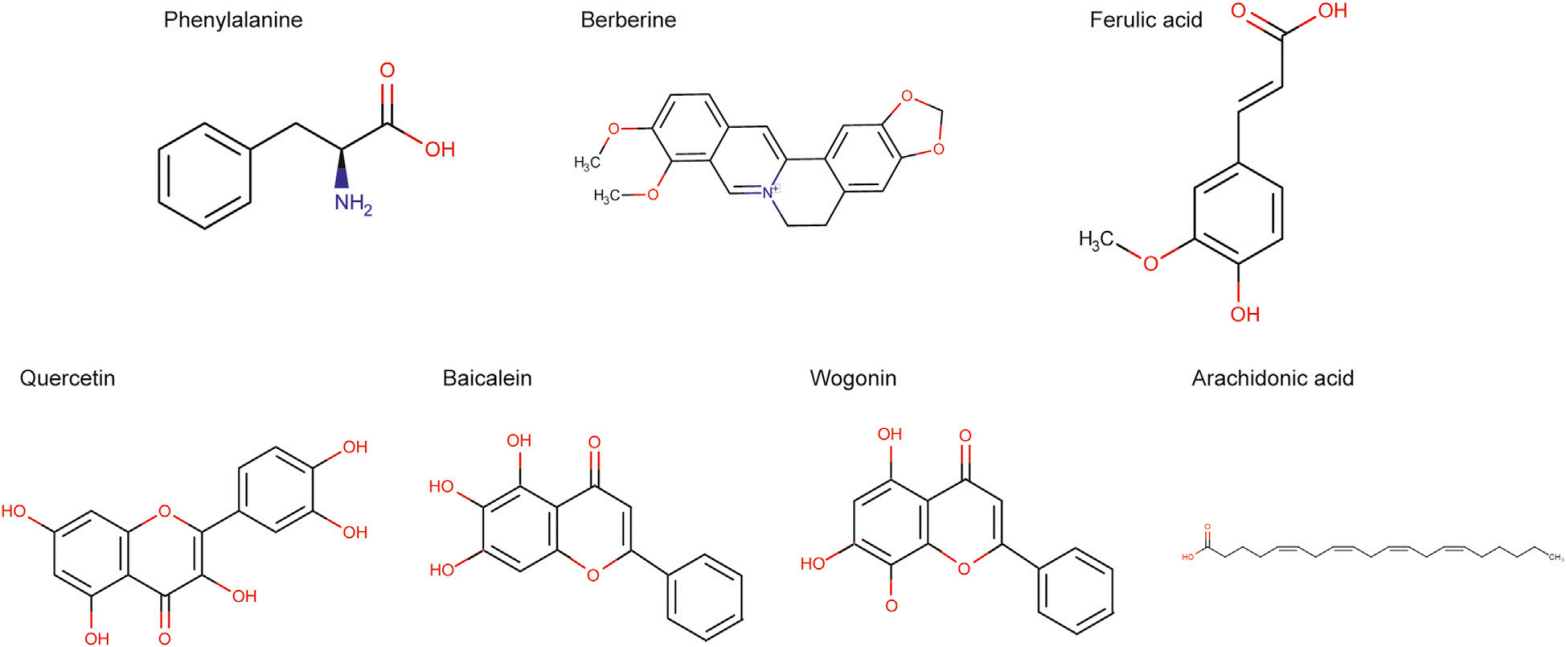
The structure of active seven compounds.

The data collected by high-resolution liquid quality were preliminarily sorted out by CD2.1 (Thermo Fisher) and then compared with database retrieval (mzCloud, mzVault, ChemSpider). The comprehensive score of seven compounds in mzCloud best match was more than 90. The structures of active seven compounds were included in Figure 7. The chromatogram collection and integration of each analyte were processed by software Xcilabur 4.1 (Thermo Fisher), and linear regression was performed with 1/x2 as weighting coefficient. All of the seven components showed good linearity (Table 5). Table 6 shows the contents of seven compounds in the sample.

**TABLE 5:** The linearities of the seven bioactive components in YDJDG determined by LC-MS/MS.

Analyte	Calibration curve	*R* ^2^
Phenylalanine	y = 259151x	1.0000
Berberine	y = −1.19209e-007 + 366053x	1.0000
Ferulic acid	y = 1539.26x	1.0000
Quercetin	y = 5284.91x	1.0000
Baicalein	y = 20047.4x	1.0000
Wogonin	y = 517664x	1.0000
Arachidonic acid	y = 56135.7x	1.0000

**TABLE 6:** The contents of seven compounds in the YDJDG.

	Sample weight (g)	Total volume of solvent (ml)	Concentration of analyte in extract of YDJDG (ng/g)						
			Phenylalanine	Berberine	Ferulic acid	Quercetin	Baicalein	Arachidonic acid	Wogonin
1	0.20359	1.017	4,723	2,203	31,218	1,020	53,619	22,049	20,492
2	0.19823	0.991	5,606	2,348	41,410	824	64,374	24,791	23,834
3	0.20102	1.005	5,599	2,267	40,560	781	65,078	25,723	25,413

## Discussion

The diagnosis and treatment plan (trial version 3) for the new coronavirus pneumonia has incorporated TCM therapy. Four days after the release of trial version 3, version 4 recommended strengthening the integration between Chinese and Western medicine, proposing the application of patent Chinese medicines during the clinical observation period, as well as determining the optimal composition and dosage of Chinese herbal medicines. In Beijing, the Beijing Ditan Hospital is the designated hospital for COVID-19. This hospital attaches great importance to the role of TCM in the prevention and control of COVID-19; it has constantly summarized and optimized treatment plans including TCM and formed an in-hospital prescription-YDJDG. The treatment cases have mild, moderate, severe, and critical disease types, and YDJDG has achieved good efficacy for all of these.

In the clinic, CD4 + T cell testing is always nonessential for COVID-19 patients. Based on data from patients with SARS in 2003–2004 (caused by SARS-CoV), and the fact that most acute viral infections result in the development of protective immunity (Sallusto et al., 2010), there is a good possibility that the level of CD4^+^ T cells, as important immune cells, has a strong relationship with COVID-19 convalescence (Peng et al., 2020). A study detected SARS-CoV-2-reactive CD4^+^ T cells in 40%–60% of unexposed individuals, suggesting cross-reactive T cell recognition between circulating “common cold” coronaviruses and SARS-CoV-2 (Grifoni et al., 2020). In this study, the clinical characteristics exhibited by COVID-19 patients, as well as treatment methods used, were subjected to Cox regression analysis. We observed that YDJDG and CD4 ≥ 660 cells/µL were independent predictors of individual proportion recovery. Moreover, after stratifying the proportion recovery based on different treatments, we further identified the applicable patients for YDJDG therapy. In the YDJDG group, the proportion recovery was significantly higher than that observed in the lopinavir-ritonavir group, for the mild/moderate disease type and CD4 < 660 cells/µL subgroups. Additionally, the mean duration of fever and pulmonary exudative lesions was significantly shorter in the YDJDG group than that in the lopinavir-ritonavir group. Although no significant difference was observed in the mean duration of positive nucleic acid detection between the two groups (*p* = 0.0729), the data showed that YDJDG demonstrated a certain inhibitory effect on the virus when compared with lopinavir-ritonavir treatment. The ingredients present in YDJDG, *Ephedrae Herba* and *Gypsum Fibrosum*, are also the main components of the *Maxing Shigan* decoction used to treat influenza. This decoction has demonstrated antiviral effects, damaging the viral surface ultrastructure, profoundly inhibiting the synthesis of viral RNA and protein, and inhibiting the entry of the influenza virus by regulating the PI3K/AKT signaling pathway (Hsieh et al., 2012). Reportedly, *Scutellariae Radix* can directly inhibit the synthesis and release of inflammatory cytokines (Shen et al., 2017). Recent reports have revealed that *Lonicerae Japonicae Flos* possesses antiviral efficacy and can inhibit inflammation. Furthermore, an extract of *Lonicerae Japonicae Flos* directly inhibited the activity of cyclooxygenase 1 (COX-1) and COX-2, as well as the expression of COX-2 protein and mRNA induced by interleukin (IL)-1β (Xu et al., 2007). The patented Chinese medicine Pudilan (PDL), containing *Scutellariae Radix*, could prevent SARS-CoV-2 from entering cells by blocking angiotensin-converting enzyme 2 (ACE2). Furthermore, it could inhibit cytokine storm by affecting the levels of IL- 6, IL-10, interferon- (IFN-) γ, tumor necrosis factor, and C-reactive protein (Kong et al., 2020). Reports have revealed that *Cimicifugae Rhizoma* could inhibit respiratory syncytial virus attachment and internalization and stimulate epithelial cells to secrete IFN-β against viral infection (Wang et al., 2012).

However, the current study demonstrates some limitations. This study was not randomized, controlled, and double-blinded; hence, an imbalance in treatment assignment could have been present, which may have affected the results of the study. However, we utilized PSM to reduce this bias. Notably, the baseline of the two groups was balanced after PSM. Additionally, the number of subjects (60 in the YDJDG and 71 in the lopinavir-ritonavir group) was low and the mechanism of YDJDG improving the prognosis of patients is not clear. Large sample sizes and mechanism are needed to verify this result in the future. Given that YDJDG is a complex mixture with a lack of clearly defined chemical profiles of the preparation, the reproducibility of this intervention needs to be considered. We tried our best to describe the herbal medicine in detail to facilitate the replication of this research in other clinical trials, including the confirmation of species sources, the description of the detailed manufacturing process, and the listing of the concentrations of the dominating compounds. The proportion of recovery was similar for the severe/critical disease type or CD4 ≥ 660 cells/µL after YDJDG or lopinavir-ritonavir therapy (*p* = 0.0660 and *p* = 0.6300, respectively), but we clarified that YDJDG treatment is more suitable for patients with mild/moderate type or CD4 < 660 cells/µL. Finally, one criterion for recovery assessment is the conversion of the result of the nucleic acid test to negative, twice consecutively (at intervals of 24 h). However, we currently lack patient data regarding whether the viral nucleic acid detection test result turned positive after discharge; hence, we plan to follow up on these patients to assess the viral nucleic acid detection results.

## Conclusion

In patients with COVID-19, YDJDG revealed effects in shortening the duration of fever and promoting the absorption of inflammatory exudates, with good safety profile. Moreover, compared with the lopinavir-ritonavir group, the YDJDG group showed a shorter recovery period for mild/moderate disease type or CD4 < 660 cells/µL. In view of the efficacy and safety, YDJDG might be considered as a candidate treatment for COVID-19. Randomized, controlled, and double-blinded trials with large sample size are needed to further solid the efficacy evidence of YDJDG for COVID-19.

## Data Availability

The raw data supporting the conclusions of this article will be made available by the authors, without undue reservation.
